# Object Detection Applied to Indoor Environments for Mobile Robot Navigation

**DOI:** 10.3390/s16081180

**Published:** 2016-07-28

**Authors:** Alejandra Carolina Hernández, Clara Gómez, Jonathan Crespo, Ramón Barber

**Affiliations:** Department of Systems Engineering and Automation, Carlos III University of Madrid, Madrid 28911, Spain; alejandracarolina.hernandez@alumnos.uc3m.es (A.C.H.); claragobla@gmail.com (C.G.); jocrespo@ing.uc3m.es (J.C.)

**Keywords:** object detection, object classification, shapes descriptors, Support Vector Machine, mobile robots, robot navigation

## Abstract

To move around the environment, human beings depend on sight more than their other senses, because it provides information about the size, shape, color and position of an object. The increasing interest in building autonomous mobile systems makes the detection and recognition of objects in indoor environments a very important and challenging task. In this work, a vision system to detect objects considering usual human environments, able to work on a real mobile robot, is developed. In the proposed system, the classification method used is Support Vector Machine (SVM) and as input to this system, RGB and depth images are used. Different segmentation techniques have been applied to each kind of object. Similarly, two alternatives to extract features of the objects are explored, based on geometric shape descriptors and bag of words. The experimental results have demonstrated the usefulness of the system for the detection and location of the objects in indoor environments. Furthermore, through the comparison of two proposed methods for extracting features, it has been determined which alternative offers better performance. The final results have been obtained taking into account the proposed problem and that the environment has not been changed, that is to say, the environment has not been altered to perform the tests.

## 1. Introduction

Robots need to have a set of capabilities that allows them to move and interact with a real environment. Among all of the skills needed, perception constitutes one of the cornerstones. The word “perception” refers to, among other things, sensory awareness. From the five different senses that humans have, vision is arguably the most important for safely moving and interacting with the world. Object detection is an important research topic in computer vision due to its wide range of applications. Great advances have been made in the past decade, especially since the work by [1].

Through many investigations, it can be seen that the techniques and methods to detect objects depend on the application environment. On the one hand, there are applications for industry, especially based on robotic arms that need the important accuracy to perform grasping and manipulation tasks. On the other hand, there are systems developed to work on mobile robots, which allow performing navigation tasks and environment categorization, among others, where accuracy is sometimes not the most important factor. This work is focused in this type of perception system.

Object perception is an essential component that the authors have reported to be the most limiting factor. Object recognition in real scenes is one of the most challenging problems in computer vision, as it is necessary to deal with difficulties, such as viewpoint changes, occlusions, illumination variations, background clutter or sensor noise. Because of this, there have been numerous methods for object recognition developed over the last few decades. Most of them concentrate on specific cases, like faces or pedestrians, with different techniques based on feature descriptors, shape descriptors, gradient-based, derivative-based, template matching, etc.

All recognition methods have in common that they require some form of representation of the object to be found. Almost all object recognition systems can be split into two successive phases: the offline phase, including the generation of the model, and the online phase, in which the constructed model is used to find the object in the search image. Thus, only the computation time of the online phase is critical considering the real-time requirement.

Numerous methods for object recognition applied to mobile robots have been developed over the last few decades. Some researchers [2,3] show the use of Speeded-Up Robust Features (SURF) [4] in object detection in mobile robots. Other methods based on bag of words (or bag of features) for object classification are presented in [5]. These methods produce good results for object detection due to the large number of descriptors extracted in each image. However, these approaches can lead to problems due to the computing power required and the delay generated while processing this amount of data. A vision system for a mobile robot should be light and have a processing speed that allows the robot to move and simultaneously detect and locate objects in the environment. In our work, two alternatives for feature extraction are compared (descriptors and geometric features). Through the tests, we demonstrate that the best performance is obtained using geometric features. The combination of a few features allows a proper discrimination of each object, and it makes the system fast and useful for mobile robots to perform navigation tasks.

On the other hand, some methods for object recognition are based on machine learning. Machine learning algorithms are organized into taxonomy, based on the desired outcome of the algorithm [6]. Common algorithm types include supervised learning, where the algorithm generates a function that maps inputs to desired outputs. One standard formulation of the supervised learning task is the classification problem: the learner is required to learn (to approximate the behavior of) a function, which maps a vector into one of several classes by looking at several input-output examples of the function. Support Vector Machine (SVM) is a useful technique for data classification [7]. A classification task usually involves separating data into training and testing sets. Each instance in the training set contains one “target value” (the class label) and several attributes (the features or observed variables). The goal of an SVM is to produce a model (based on the training data) that predicts the target values of the test data given only the test data attributes. Some researchers [8] use SVM as a classification algorithm combined with others. They present a system that use convolutional neural networks. In the same way, the work in [9] propose a framework that can detect objects and estimate their poses simultaneously by matching one of the 3D models in a database.

Among the most important stages of an object detection system is the image segmentation [10]. There are many image segmentation techniques, some of them are based on color [11,12]. In [11], a method is proposed that uses depth segmentation techniques and processes the color and depth images provided by the Kinect sensor. In the process, a color segmentation to detect only red chairs is performed, creating an initial mask allowing objects of the same color to be extracted. One disadvantage of this type of method is that the segmentation based on color is susceptible to changes in illumination. In our work, a combination of different segmentation techniques that do not involve color information is implemented; in this way, problems related to changes in lighting are eliminated, making our algorithm more general to detect different objects, irrespective of color. Other approaches use 3D information for point cloud segmentation, as the work of [13]. They present an approach to localize planar furniture parts in 3D range camera data. The segmentation method uses shape information to detect chairs with elliptical shapes. This approach is designed for specific objects (elliptical chairs), so generalizing the algorithm to other objects can be difficult. In our work, we propose to use the geometric information without focusing on a particular shape, but combining features that distinguish one object from another in a better way.

Otherwise, there are region growing techniques, such as watershed [14]. There are many research works in the literature that evaluate and compare the performance of watershed segmentation, for example the work of [15] for binary images with different distance transforms. Likewise, a method based on the watershed transformation combined with a fast algorithm based on the topological gradient is presented in [16]. In order to avoid an oversegmentation, they propose to adapt the topological gradient method obtaining good results for image segmentation.

Finally, we propose an object detection system integrated into a real mobile robot able to detect objects present in usual human environments. In our case, the system must detect and locate common objects in indoor environments. These environments have not been altered to perform the tests, i.e., lighting conditions, objects positions, among others, have not been controlled. It is important to mention that the detected objects generate events that will be used by a topological navigation system, where the only information needed is that which ensures that the event is detected.

Segmentation techniques based on contours’ extraction and the watershed algorithm to segment the selected objects have been implemented. The implemented feature extraction techniques are based on geometric information. Furthermore, the proposed detection system is based on SVM as the classification algorithm. Through the combination of all of these methods and techniques, a vision system has been developed that ensures a proper processing speed. This is a key factor to guarantee that the robot moves and simultaneously detects and locates objects in indoor environments.

## 2. Proposed System

In this work, a vision system to detect objects considering usual human environments, which means indoor environments without any modifications, and able to work on real mobile robot is developed. In this section, a general explanation of the structure of the system and its component parts is presented.

### 2.1. General Approach of the Proposed System

**Figure 1:**
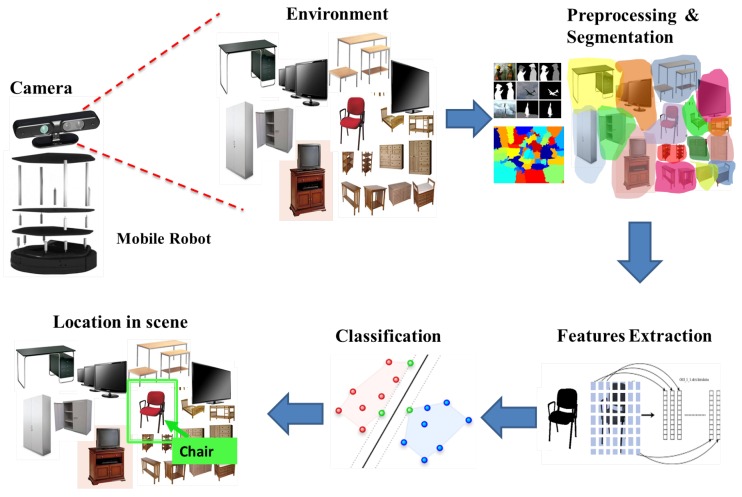
General approach of the proposed system.

The proposed system is integrated into a real mobile robot, and a camera with specific characteristics as a sensor to capture images is used. The idea is to capture real-time images of indoor environments, process them, extract the features of the objects in the scene, then use a classifier to detect the object to finally locate them in the original scene. In Figure 1, the general approach of the system is shown.

**Figure 2:**
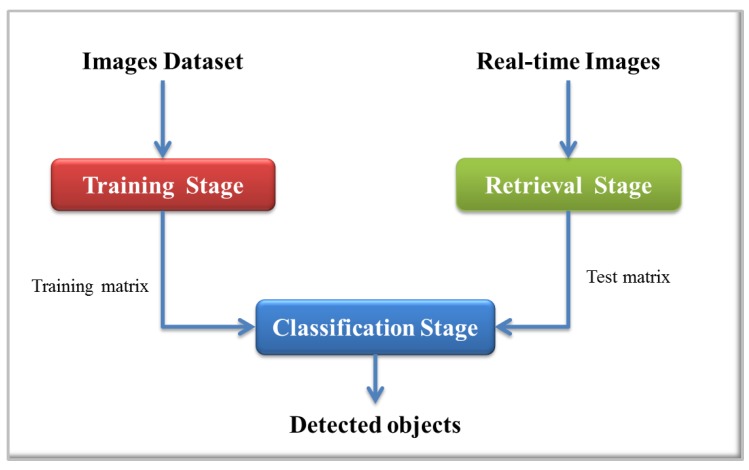
General outline of the proposed system.

The proposed detection system is based on Support Vector Machines (SVMs) as the classification algorithm to detect objects in indoor environments. As input to this system, RGB and depth images are used. The implemented process is depicted in Figure 2, which is divided into three main stages: an offline stage to train the classifier and two online stages, one for the preprocessing of the retrieved images and the other where the object classification process is performed.

The first stage covers the whole process since the creation of the dataset of images, initial preprocessing, segmentation, to feature extraction and, finally, the training process. In the second stage (online), obtaining the real-time image, preprocessing, segmentation and feature extraction are included. Finally, in the classification stage, filtering of the results from the classifier and the location of objects in the image are performed.

### 2.2. Detailed Description of the Proposed System

**Figure 3:**
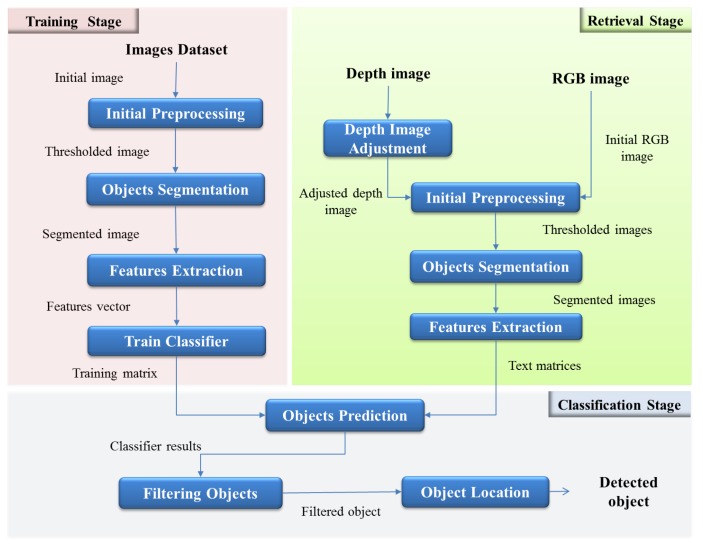
Detailed diagram of the proposed system.

In Figure 3, the detail of each stage of the system and its corresponding interaction are shown.

The process begins with the preparation of images to train. To do this, several images of selected objects to detect are taken with the chosen camera. Next, each image is preprocessed in order to obtain a thresholded image, which is the input to the next step, segmentation. Image preprocessing techniques and morphological transformations are used to get an image without noise, with better contrast and highlighted regions of interest. Then, the thresholded image is used to perform the segmentation process.

As is explained in the next section, for each selected object to be detected, different segmentation techniques are applied to each image. The next step is to find the features of the object. In this work, two alternatives to extract features of the objects are explored: geometric shape descriptors and bag of words are implemented. To complete this first stage, training matrices are created to train the classifier. In this step, the parameters of SVMs are defined.

In the second stage, the retrieved image preprocessing is performed. This process is conducted entirely online, and real-time images in different formats, color RGB and depth images are taken. It is necessary to make an adjustment process for depth images to remove noise and invalid pixels. Then, both depth and RGB images are sent to the preprocessing step, which is similar to the preprocessing of the training stage. Subsequently, the segmentation is applied to both images, and features from each isolated object are extracted. The final goal of using different types of segmentation and feature extraction methods is to evaluate functionality and to compare the results.

The final stage consists of predicting the objects in the real-time images. For this, test matrices are created with features extracted, and SVMs use this along with training information to classify the object. Next, filters are applied to make a decision according to the results of the SVMs. Finally, a process to locate the detected object in the scene is performed. The center of mass of the object is calculated, and depth information is used to determine the distance and angle from the center of the camera. This information is encapsulated and prepared to be sent to other systems with the objective to contribute with navigation tasks and high level tasks concerning places’ categorization and semantic navigation.

## 3. Image Preprocessing: Training Stage

Building an object detection system requires a process called training, which can be described as a machine learning about the object that will be detected in images. The training consists of finding the rules that best classify the object and combining them to form each stage of the detector. This stage performs the tasks needed to train the selected classifier. The main goal during this stage is to obtain a database containing the model of the objects to be detected, which will be used to train the selected classifier.

### 3.1. Images Dataset

One of the aims of this work is to develop an object detection system able to work in usual human environments. In the same way, this work is intends to be useful for semantic navigation systems that allow the robot to relate what it perceives to the place in which it is located. This way, an environment model is managed based on the objects and the concepts that they represent [17]. Thus, object recognition is another base on which a semantic navigation system may rely [18].

**Figure 4:**
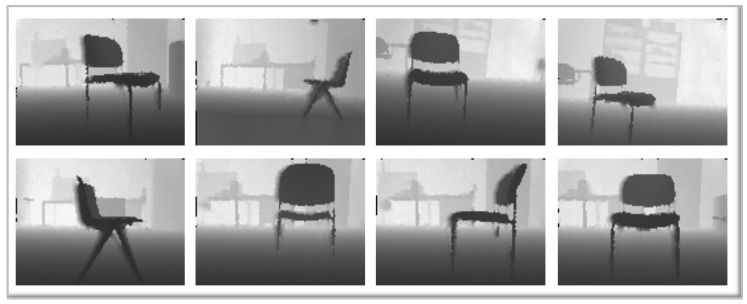
Dataset of Class 2: chairs.

Taking into account the above, to create the training data, as a first step, a dataset of objects present in usual human environments has been created. The dataset contains RGB and depth images acquired by an ASUS Xtion pro Live sensor (Artcreation 3d Technology Limited, Hong Kong, China), which was mounted on a mobile platform at a height of 40 cm. The selected environment is a laboratory of the Carlos III University of Madrid, and data have been collected from three common objects present in laboratories: chairs, closets and screens, including TV screens and computer screens. Each type of object represents a class. Thereby, the system initially consists of three classes, Class 1 for closets, Class 2 for chairs and Class 3 for screens. Figure 4 shows an example of the dataset of images for the chair object.

### 3.2. Initial Preprocessing

This step consists of preparing each image of the dataset to the segmentation process. For this, the following techniques have been applied:

- Equalization: to improve the contrast in an image, in order to stretch out the intensity range.
- Morphological operations: erosion and dilation to remove small objects from each image while preserving the shape and size of larger objects in the image.
- Gaussian filter: to “blur” images and remove details and noise. A Gaussian blur effect is typically generated by convolving an image with a Gaussian function.
- Thresholding: to separate out the regions of the image corresponding to initial interest points, from the regions of the image that correspond to the background.

**Figure 5:**
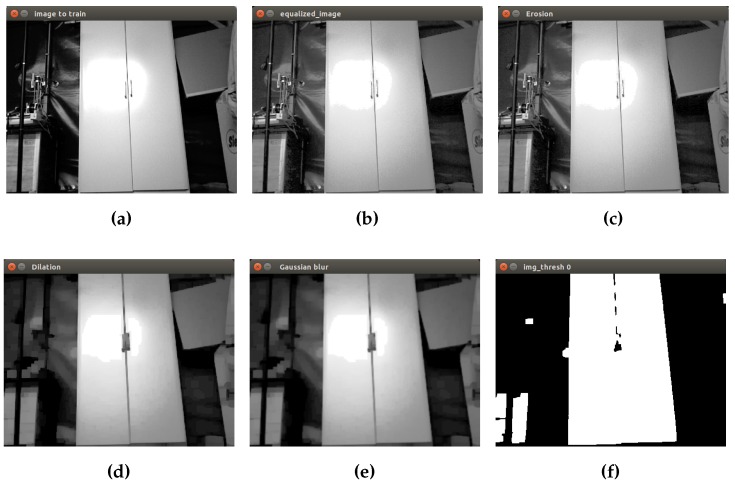
Initial preprocessing of the closet. (**a**) Initial grayscale image; (**b**) equalization; (**c**) erosion; (**d**) dilation; (**e**) Gaussian filter; (**f**) binary image.

Finally, a binary image as input to the next step is obtained. In Figure 5, the process to prepare the closet object is shown. The first image corresponds to the initial grayscale image Figure 5a. Then, equalization, erosion and dilation are applied to remove small objects in the scene. The Gaussian filter is applied in order to reduce image noise and details using a 3 × 3 kernel value. Finally, in the last picture, the isolated closet can be seen by converting the grayscale image into a binary image.

This process is applied to all of the images of the dataset. Different kernel and threshold values are used in order to obtain an initial discrimination of the objects present in the scene.

### 3.3. Object Segmentation

There is not a single segmentation method that can be considered good for different images; every method is not equally good for a particular type of image [19]. Algorithm development for one class of images may not always be applied to other class of images. Hence, there are many challenging issues, like the development of a unified approach for image segmentation that can be applied to all types of images; even the selection of an appropriate technique for a specific type of image is a difficult problem. Thus, in spite of several decades of research, there is no universally-accepted method for image segmentation, and therefore, it remains a challenging problem in image processing and computer vision [20]. For these reasons, in this step, different segmentation methods have been applied to each kind of object in order to isolate as much as possible the object in the image.

#### 3.3.1. Chair Object Segmentation

The image format used to process the chairs is the depth image. Hence, the first segmentation process is based on contours’ extraction. OpenCV offers a function called findContours, which retrieves contours from the binary image using the algorithm proposed by [21]. This function uses as input the following parameters: input image, output vector contours, hierarchy vector, contour retrieval mode and contour approximation method.

One of their outputs is the hierarchy, a vector that contains information about the image topology. It has as many elements as the number of contours. For each *i*-th contour contours[i], the elements hierarchy[i][0], hierarchy[i][1], hierarchy[i][2] and hierarchy[i][3] are set to zero-based indices in contours of the next and previous contours at the same hierarchical level, the first child contour and the parent contour, respectively. If for the contour *i*, there are no next, previous, parent or nested contours, the corresponding elements of hierarchy[i] will be negative [22]. In summary, the main idea in this first segmentation is to find only the external contour that according to the hierarchy contour concept meets the following conditions:

- the contour has a child. (hierarchy[k][2]>=0)
- the contour has no parent. (hierarchy[k][3]<0)

**Figure 6:**
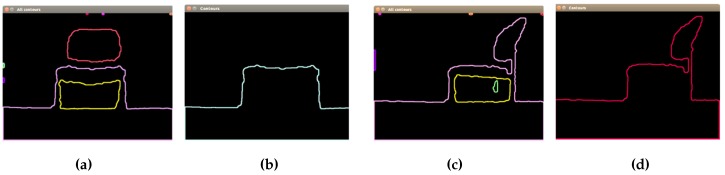
Segmentation of chairs. (**a**) Chair 1: extraction of all contours; (**b**) chair 1: external contour; (**c**) chair 2: extraction of all contours; (**d**) chair 2: external contour.

This proposed segmentation process applied to two different chairs can be seen in Figure 6. First, contours’ extraction is performed, using as input the thresholded image obtained in the previous step. Then, the concept of hierarchy is applied, finding contours with a child and contours without a parent.

#### 3.3.2. Closet Object Segmentation

Among the most important physical features that define the closets, the size (large) and the presence of door handles have been considered for developing the segmentation method. The method is also based on contour extraction, but by following these steps:

- Find out the biggest contour by comparing the area of each contour in the image, in order to eliminate small objects that are not of interest to this process.
- Create a mask with the biggest contour to crop image. A mask is a filter. The concept of masking is also known as spatial filtering. By applying a mask on an image, the pixels of the input image whose corresponding pixels in the mask are true are copied into a new image. The rest of the pixels in the new image are set to zero.
- Find the contour of the door handle. Applying the hierarchy contour concept, the presence of a child contour in the biggest contour is considered a door handle.

**Figure 7:**
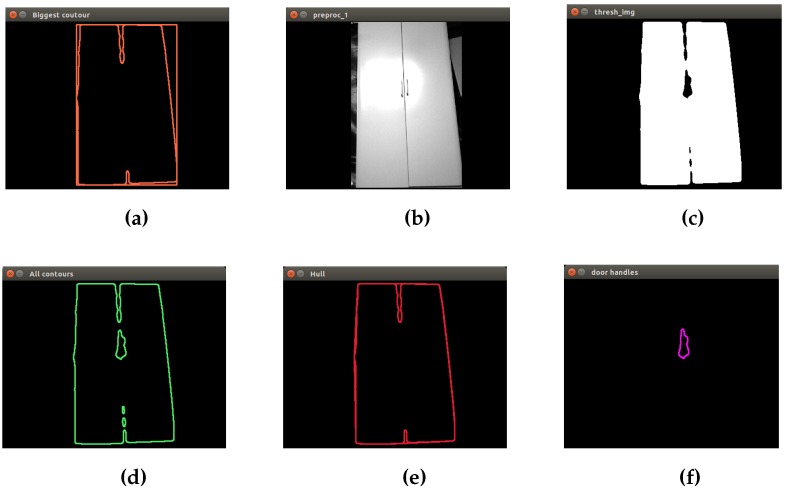
Closet segmentation: second algorithm. (**a**) The biggest contour of the image; (**b**) result of applying the binary mask in the input image; (**c**) thresholding operation; (**d**) extraction of all contours; (**e**) extraction of the external contour; (**f**) extraction of the possible door handles.

In Figure 7, the result of the segmentation method is shown.

#### 3.3.3. Screen Object Segmentation

**Figure 8:**
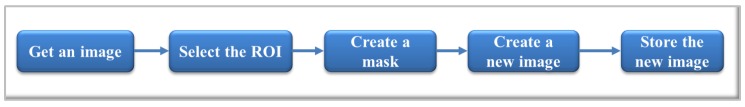
Diagram of the process for ROI segmentation.

Following the idea of trying different methods of segmentation, to segment the screen object, another technique has been applied. In this case, a segmentation algorithm based on the selection of regions of interest (ROI) has been created. A region of interest is a region of the image where one is interested, and the processing is done on that region. A small portion of the image is taken, and the processing is done in that part instead of processing the whole image [23]. In Figure 8, the diagram of the process developed is shown.

**Figure 9:**
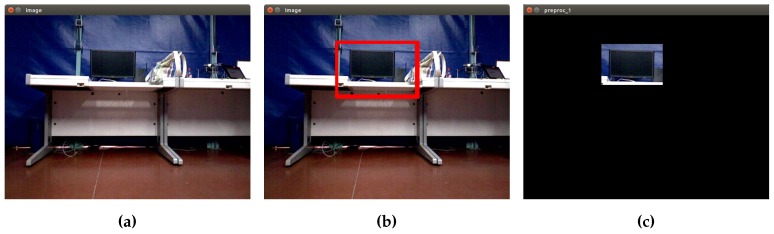
ROI segmentation process applied to a screen object. (**a**) Original image; (**b**) interest region; (**c**) mask.

The process begins by getting an image from the camera or from a file. Then, the region of interest is selected by drawing a rectangle with the mouse enclosing the desired area. With this bounding box, a mask is created, isolating a sub-section of the current image. From this, a new image with the specific region is created and finally stored. This method allows one to work with certain regions of images, improving accuracy in the segmentation process. In Figure 9, the results of applying this method to segment a screen object are shown.

### 3.4. Feature Extraction

This step is one of the most important of all of the process, because the features that define an object are determined, in addition to establishing what differentiates one object from another. Each object has physical features that define it. For the closet object, one of the most important features that distinguish it from other object with the same size is the presence of door handles. In the case of a chair, the legs are important to define it. Screens are very difficult to discriminate, because their shape is very common in other objects in indoor environments. In this work, two alternatives for extracting features are presented: one, through shape features, and another, through image descriptors.

#### 3.4.1. Shape Features

**Figure 10:**
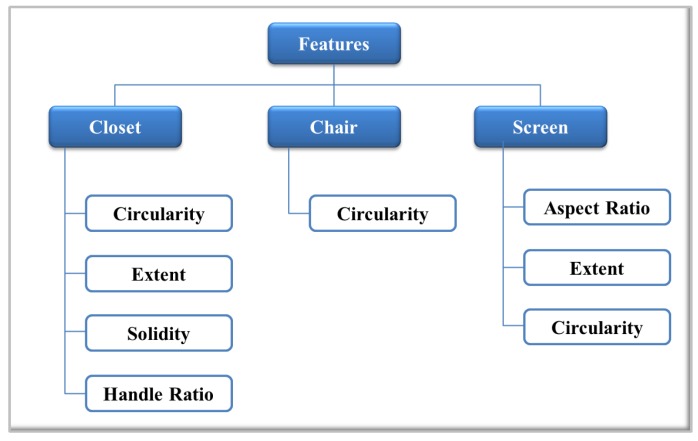
Features extracted for the selected object.

In this approach, the idea is to find features invariant to rotation, translation and size. According to the selected objects, different geometric features have been chosen. The combination of these features allows one to discriminate shapes and must be able to define and differentiate each object. In Figure 10, a diagram with the selected features for closets, chairs and screens is shown. In the case of the closet object, solidity, extent and circularity are relative to the shape of the object. Additionally, to ensure a better differentiation of the object, a fourth feature, called handle ratio, has been created. Handle ratio is a feature that represents a value defined between 0.8 and 0.9 when the door handle appears in the image. If the door handle can not be detected, the ratio assigned is between 0.0 and 0.1.

For the chair object, major efforts have been made to reach a better segmentation; therefore, only one feature (circularity) is needed to define this object. Regarding the screen object, three features, circularity, extent and aspect ratio, have been chosen. One of the most important features of screens is the aspect ratio. With each set of features, a training matrix is created. Each row corresponds to one image. Each element in that row corresponds to one feature of the class. Additionally, a label vector with the number of the corresponding class is created.

#### 3.4.2. Bag of Words

The idea is treating image features as words. The SURF extractor and descriptor is used. For this process, OpenCV has some very useful libraries that have been used. The steps followed to build a Bag of Words (BoW) with SURF features are:

1. Obtaining the set of bags of features.
  - Extract the SURF feature points of all of the images in the set.
  - Obtain the SURF descriptor for each feature point that is extracted from each image.
  - Cluster the set of feature descriptors for the amount of bags defined.
  - Obtain the visual vocabulary.
2. Obtaining the BoW descriptor for the given images.
  - Extract SURF feature points of the given images.
  - Obtain SURF descriptor for each feature point.
  - Match the feature descriptors with the vocabulary created in the first step.
  - Represent images by the frequencies of visual words, extracting the histogram in the form of visual words for each image.

### 3.5. Training the Classifier

In this step, each training matrix is used to train the classifier. The parameters of support vector machine are defined, and training information is stored in an XML file. Table 1 shows the parameters selected for SVMs used in the proposed system. The SVM type is the type of SVM formulation. Using one class, all of the training data are from the same class, SVM builds a boundary that separates the class from the rest of the feature space. If multiple classes are working on one machine, C-Support Vector Classification (SVC) is used, because it allows imperfect separation of classes with the penalty multiplier C for outliers. The kernel type is the type of SVM kernel. In this case, a linear kernel has been chosen. It is the fastest option.

Gamma is the parameter *γ* of a kernel function; nu is the parameter *ν* of an SVM optimization problem. Termination criteria of the iterative SVM training procedure, which solves a partial case of the constrained quadratic optimization problem, can be tolerance and/or the maximum number of iterations. In this case, both are specified. These parameters are stored in an object of the class *CvSVMParams*. Finally, all of this information is used by the SVMs to train the classifier and, later, to perform the object prediction.

## 4. Image Preprocessing: Retrieval Stage

### 4.1. Depth Image Adjustment

Initially, through the subscription to Robot Operating System (ROS) topics *camera/depth/image* and *camera/rgb/image_color*, real-time depth and RGB images are obtained. Topic *camera/depth/image* contains a 2D depth matrix containing point distances in meters encoded as a 32-bit float. In this case, the sensor used is an ASUS Xtion pro Live that operates within a range of distances from 0.45 m to 5.7 m. Converting depth values to the range of an uchar (eight bits) is necessary. Depth images of ASUS Xtion pro Live suffer from the problem of poor accuracy caused by invalid pixels, noise and unmatched edges [24]. The non-finite value (*NaN*) denotes an invalid depth. For this reason, several techniques have been applied to improve the quality of the image:

1. Convert the ROS depth image into a OpenCV image: this is possible with the following instructions:
  cv_bridge::CvImageConstPtrcv_ptr
  cv_ptr=cv_bridge::toCvShare(msg)
2. Convert depth values to the grayscale range (values between zero and 255).
3. Remove invalid pixels (*NaN*): the inpaint function from OpenCV is used. The function reconstructs a selected image area from the pixel near the area boundary.

**Figure 11:**
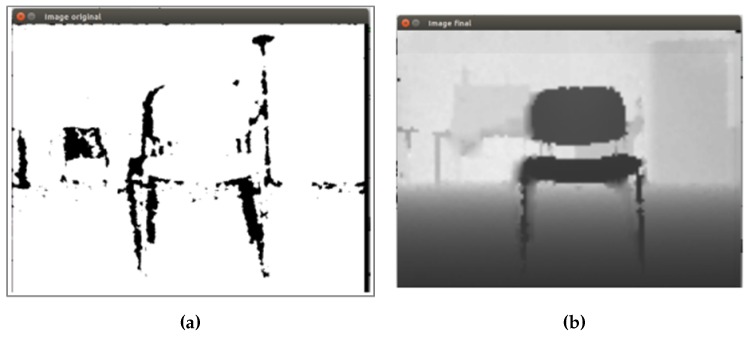
Example of depth image adjustment of the chair object. (**a**) Original depth image; (**b**) enhanced depth image.

It is important to mention that all images are resized to 70% to improve the execution time of the algorithm. In Figure 11, an example of depth image adjustment is shown.

Figure 11a shows the original depth image of a chair object obtained from the sensor. The black regions represent the invalid pixels, that is the sensor is not able to process these distances in the image. Then, after applying the proposed method, an enhanced image (Figure 11b) without invalid pixels and with a better definition, needed to continue to the next step, can be seen.

### 4.2. Initial Preprocessing

**Figure 12:**
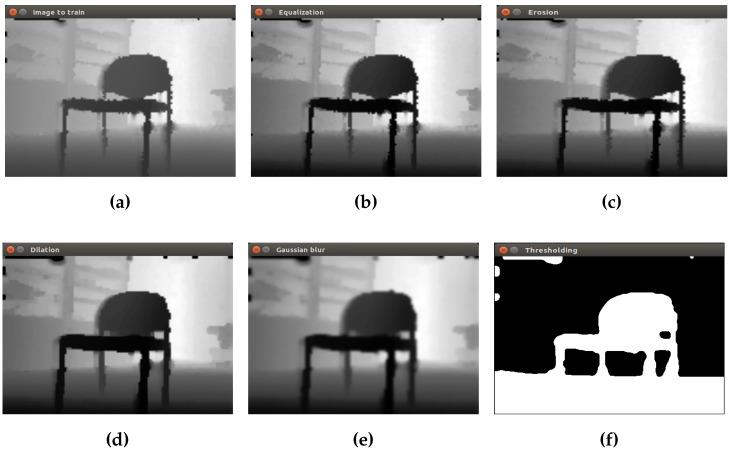
Initial preprocessing of the depth image in real-time. (**a**) The initial image; (**b**) equalization; (**c**) erosion; (**d**) dilation; (**e**) Gaussian filter; (**f**) threshold operation.

Both the RGB image and depth image must go through an initial preprocessing to remove details and noise, highlighting regions of interest, in order to prepare the images for the segmentation process. The method used is the same described in the training phase. The initial preprocessing applied to depth image in real-time can be seen in Figure 12.

**Figure 13:**
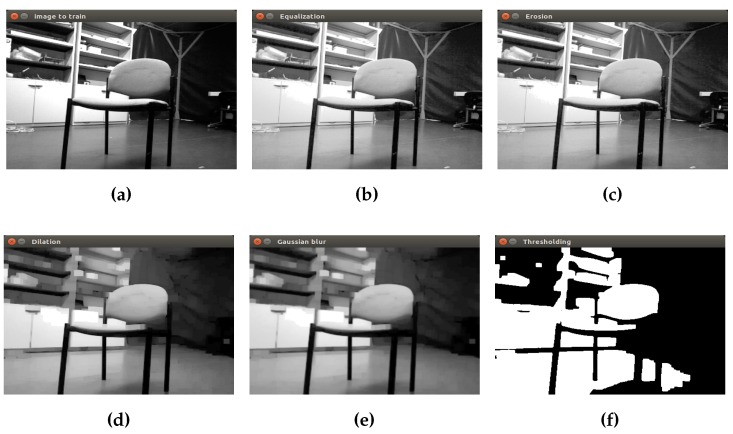
Initial preprocessing of the RGB image in real-time (chair object). (**a**) The initial image; (**b**) equalization; (**c**) erosion; (**d**) dilation; (**e**) Gaussian filter; (**f**) threshold operation.

**Figure 14:**
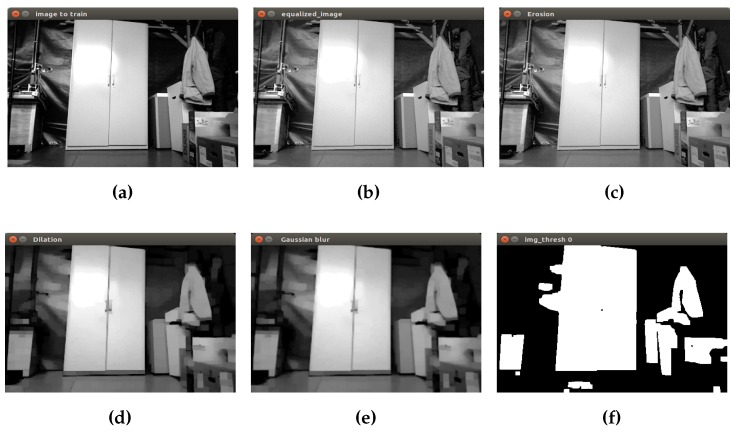
Initial preprocessing of the RGB image in real-time (closet object). (**a**) The initial image; (**b**) equalization; (**c**) erosion; (**d**) dilation; (**e**) Gaussian filter; (**f**) threshold operation.

The same process is applied to the RGB images, as can be observed in Figure 13 for a chair object and in Figure 14 for a closet object.

This process is applied to each captured image by the camera (depth and RGB format). Different kernel and threshold values are used depending on the image type, in order to get the first discrimination of the objects in the scene.

### 4.3. Objects Segmentation

**Figure 15:**
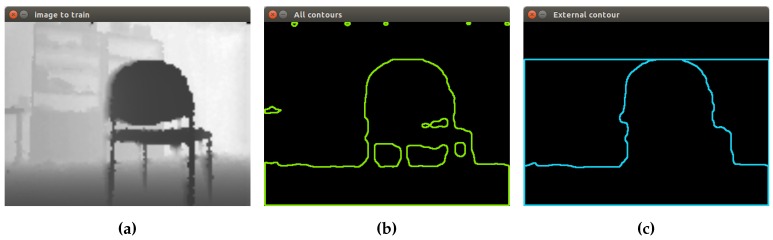
Segmentation of the depth image in real time. (**a**) Preprocessed depth image; (**b**) extraction of all contours; (**c**) external contour of a chair.

**Figure 16:**
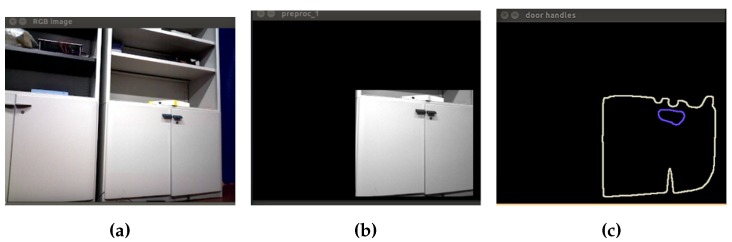
First real-time RGB image segmentation. (**a**) Original image; (**b**) result of applying the binary mask in the input image; (**c**) extraction of the possible door handles.

Segmentation is applied to both the depth image and the RGB image as follows:

- For depth images, the segmentation method for the chair object based on contour extraction is performed. Basically, it is based on the contour extraction method described in the training stage. In Figure 15, the segmentation method applied to the preprocessed depth image is shown. As already mentioned, this method is based on the concept of hierarchy, and in the case that the sensor is capturing a chair in the image, the application of the method should result in a possible contour of a chair.
- For RGB images, two segmentation processes are performed. The objects to detect, using this type of image, are closets and screens, because more details are needed. First, the same techniques applied to training images are used for real-time RGB image, in the case that the object is a closet. In Figure 16, the first RGB segmentation is shown.
- A second segmentation process in order to isolate screen objects in the image is performed. In the training stage, an algorithm based on the selection of regions of interest (ROI) has been applied. For real-time images, this is not possible, so a technique based on region growing, called watershed [25], is implemented. First, the RGB image is converted to grayscale. Then, a threshold operation is applied to obtain a binary image. With this image, morphological opening is applied to remove noise. The next step is to get the distance transformation and to normalize the transformed image in order to display it. A re-normalization to zero to 255 for further calculations is performed. Subsequently, the contours of markers are calculated, and watershed with the markers as seeds for each segment is applied. Finally, a color tab for coloring the segments is created, and different colors for each segment are assigned.

**Figure 17:**
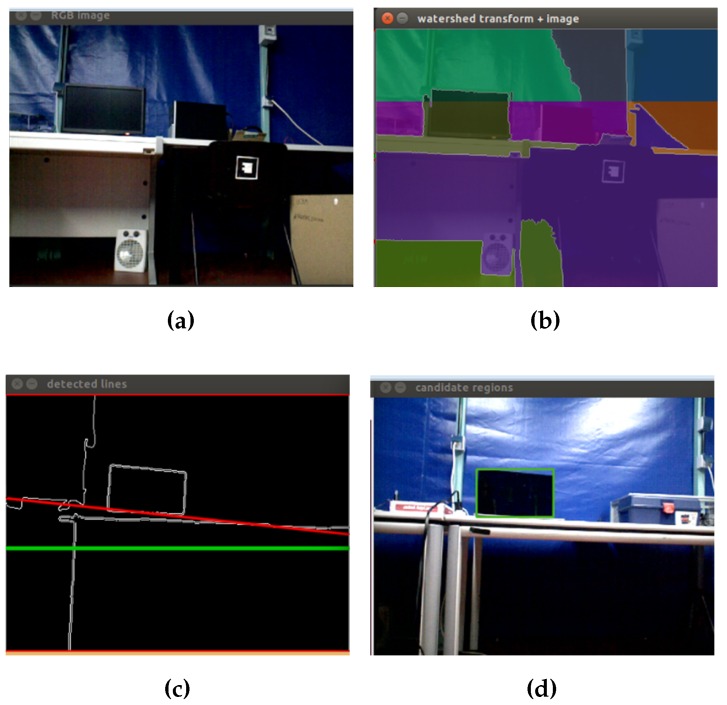
Screens segmentation algorithm. (**a**) Original image; (**b**) application of the watershed algorithm; (**c**) application of the Hough transform; (**d**) contours extracted from the regions.

With the segments created, the next step is to find regions away from the floor. For this, the idea is to get all of the horizontal lines present in the image and to select the line that meets certain conditions that allow the filtering of regions that may possibly be screens on a table. For this, Hough transform is used. Therefore, knowing the range of angles in which the lines are present in the image, a conditional statement to filter out the lines detected in the desired angle range is implemented. Thereby, vertical lines are removed, and the best horizontal line is selected. Thus, regions near the selected line are chosen. Finally, contours are extracted from the selected region. Figure 17 shows the application of the watershed algorithm and the Hough transform in real-time images.

### 4.4. Feature Extraction

**Figure 18:**
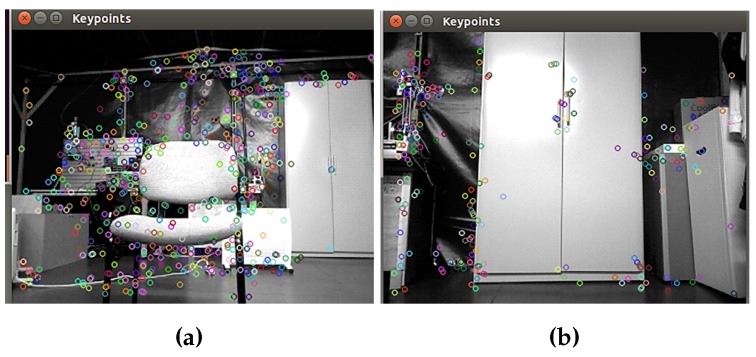
Descriptors extracted in real-time images. (**a**) Chair descriptors; (**b**) closet descriptors.

After the segmentation process, features from each type of image are extracted, considering the important physical features of the selected objects to detect. In the training stage, two alternatives for extracting features, one through shape features and another through image descriptors, have been mentioned. As for the first alternative shape features, the same features are extracted for both depth and RGB segmented images. On the other hand, using image descriptors, the SURF extractor and descriptor are used, and a bag of words with SURF features is created. In Figure 18, the colored circles represent the descriptors extracted with the bag of words method. When a chair is captured in the scene, more descriptors are generated, which does not occur when there is a closet in the image.

After the segmentation process, test matrices are created, and all calculated features are stored in them. This completes the second stage, and the created matrices become the input to the classification stage, the last in the proposed system.

## 5. Classification Stage

### 5.1. Object Prediction

The classification method used is support vector machine, which is primarily a classifier method that performs classification tasks by constructing hyperplanes in a multidimensional space that separates cases of different class labels. In this approach, a set of binary classifiers is trained to be able to separate each class from all others. Then, each data object is classified to the class for which the largest decision value was determined. To perform the classification process, OpenCV offers a library called SVM. This implementation is based on [26]. The method predict is used to classify an input sample using a trained SVM. The function used for the classification has two parameters, *sample*, which is the input sample for prediction, in this case each test matrix, and *returnDFVal*, which specifies the type of return value. With this approach, three SVMs for the prediction process, one against all [27], are used. For this, three test matrices have been created in the retrieval stage, a matrix with the features extracted from the depth image and two matrices with the features extracted from segmented RGB images (one for closets and another for screens). For each SVM, the result variable returns a number from 1 to 3, where 1 represent if the detected object is a closet, 2 if the object is a chair and 3 if it is a screen. When three SVMs are used, three different results are generated after the prediction process.

### 5.2. Filtering Objects

This process allows one to determine which is the final result and what object has been detected. The inputs to this process are the classification results obtained in the previous step. With the results achieved simultaneously from each SVM, different combinations are generated. Table 2 shows all possible combinations of the outputs of the three SVMs.

As can be seen, for each SVM, 1 represents the presence of the object and 0 the absence of it. To obtain the final result of the detected object, a filter based on conditional statements is applied. There are simple combinations, such as Cases 4, 6 and 7, where the result is direct and corresponds to the outcome of the classifier. Case 8 is an invalid result, that is, any object has been detected in the image. In Cases 1, 2 and 3, the applied rule is based on accuracy values obtained in the preliminary experiments to test the detection system in each object separately. These tests, which are explained in the next section, show that the best model accuracy is for the closets, followed by chairs and with the lowest value, the screens. Therefore, whenever more than one SVM returns 1, including SVM 1 (which is used to test the closet objects), the definitive result will always be closet. In Case 5, considering the same principle, SVM 2 and SVM 3 return 1; thus, the final result is always chair, since it is the second object with the highest model accuracy.

### 5.3. Object Location

With the final results obtained, the next step is the location of the detected object in the original image. First, the segmented image of the object is used, and the center of mass is calculated. This information is used to show in the image, in the point of the center of mass of the object, the name of the detected object. With the final results of each approach, the next step is the location of the object detected in the original image. To determine the distance to the detected object relative to the center of the camera, the coordinates of the center of mass are obtained from the RGB image to get the corresponding distance in the depth image. For the location of the detected object, not only distance is necessary, but also the orientation relative to the center of the camera. Computing the angle only requires simple linear interpolation. For this, the resolution of the image and the angle of view of the camera are required. The process is the following:

1. Knowing the field of view (FOV) of the camera, 58° H, 4° V, 70° D (Horizontal, Vertical, Diagonal), the diagonal FOV is taken.
2. The resolution of the camera is 640 × 480 pixels, but for all images, the size has been reduced by 30%, in such a way that the real resolution for this project is 448 × 336 pixels.
3. With these premises, it is assumed that the camera with a resolution of 448 × 336 pixels covers a 70-degree angle of view across the diagonal.
4. Using trigonometry, the number of pixels among the diagonal is calculated. Then, an approximation of the pixels per degree can be obtained.

**Figure 19:**
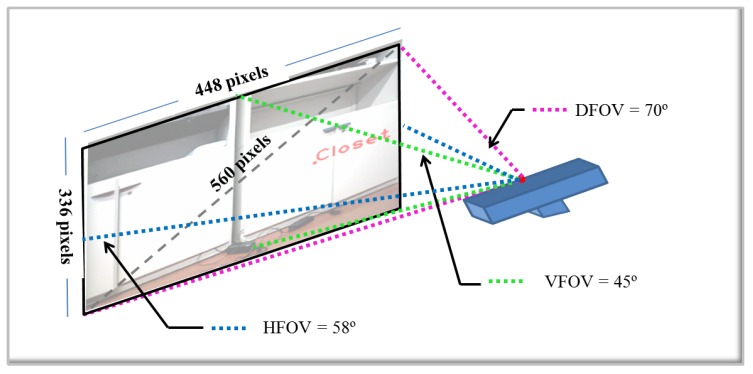
Calculation of the orientation angle of the detected object.

In Figure 19, a diagram that illustrates the process is shown.

**Figure 20:**
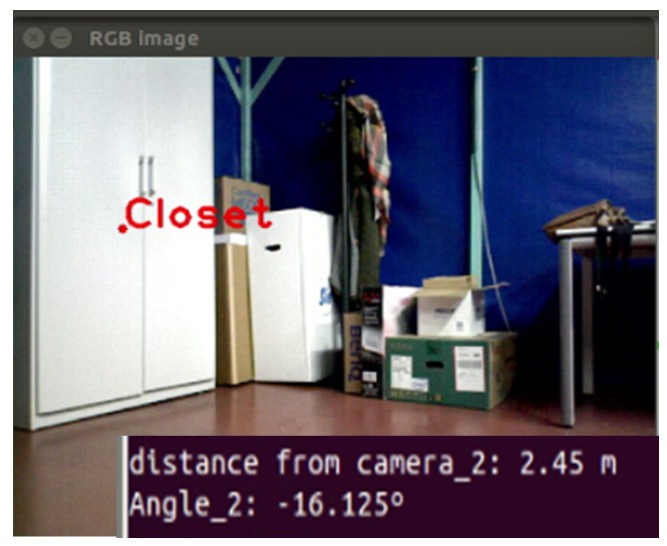
Example of the location of a detected closet.

According to the calculations, there are 560 pixels among the diagonal. That means each pixel represents 0.125 degrees per pixel. The final result about the location of the detected object is displayed in Figure 20.

Finally, this information is encapsulated in an ROS message. The message structure contains the name of the detected object, the distance from the center of the camera and the orientation angle. The name of the detected object provides semantic information that supports higher-level reasoning and can be used for navigation decisions.

## 6. Experimental Results

The aim of the following experiments is to demonstrate the usefulness of the developed system for the detection of the selected objects: chairs, closets and screens. Furthermore, a comparison between the results obtained by applying the techniques of segmentation and feature extraction presented in this work is performed. The idea is to determine which alternative offers better performance taking into account the proposed problem and the environment of the application. The advantages and disadvantages of each of them are presented. Finally, an integration test with a navigation system is performed to evaluate the real-time functioning of both systems together.

### 6.1. Work Environment

To carry out the experiments, the environment chosen for testing is a laboratory. It is important to mention that the environment has not been changed, guaranteeing the naturalness of the place. The robot selected for the implementation is a platform called TurtleBot. To build the detection system, a camera ASUS Xtion pro Live [28] as a sensor for detection is used. In order to guarantee the hardware and software integration, the proposed detection system is developed under the Robot Operating System (ROS) [29], using the C++ programming language and OpenCV libraries [30] for image processing.

### 6.2. Testing Methodology

To carry out the performance evaluation of the proposed system, the concept of the confusion matrix is used. A confusion matrix [31] of size *n* × *n* associated with a classifier shows the predicted and actual classification, where *n* is the number of different classes. Table 3 shows a confusion matrix for n=2, whose entries have the following meanings:

- *a* is the number of correct positive predictions.
- *b* is the number of incorrect negative predictions.
- *c* is the number of incorrect positive predictions.
- *d* is the number of correct negative predictions.

In the confusion matrix, rows indicate original rates, and columns indicate outputs from the classifier. The diagonals indicate the true classifications; the rest are false classifications [32]. Several measures can be obtained from this matrix. In this work, accuracy, misclassification, sensitivity and specificity are used as follows:

The accuracy is the proportion of the total number of predictions that were correct. It is determined using the equation:

(1) $$\frac{a+d}{a+b+c+d}$$

The misclassification rate is the proportion of the total number of predictions that were incorrect. It is determined using the equation:

(2) $$\frac{b+c}{a+b+c+d}$$

The recall or true positive rate (sensitivity) is the proportion of positive cases that were correctly identified, as calculated using the equation:

(3) $$\frac{a}{a+b}$$

The true negative rate (specificity) is defined as the proportion of negatives cases that were classified correctly, as calculated using the equation:

(4) $$\frac{d}{c+d}$$

## 7. Preliminary Tests

Several tests of the proposed system for each type of object (chair, closet and screen) are individually performed. The idea is to determine, from the individual results, the improvement points to implement in the integrated system. In this test, geometric shape descriptors are used as the method for feature extraction. Likewise, the camera is integrated into TurtleBot, and the procedure consists of moving the robot by teleoperation around the area of the test and taking frames to analyze the results.

In the case of chairs, 70 frames were taken, divided into 43 frames with different types of chairs in different positions and 27 frames with other objects of the selected environment. Table 4 shows the confusion matrix of this test. The evaluations of this test can be seen in Table 5, where the true positive rate is equal to 65.12% and the model accuracy is 71.45%.

**Figure 21:**
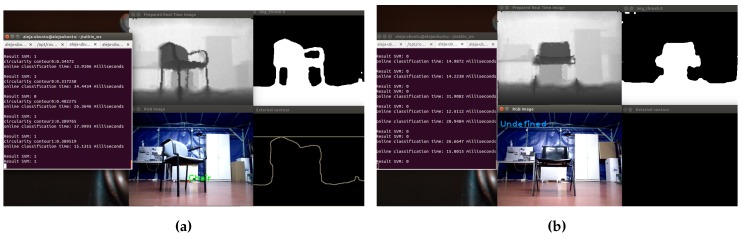
Example of the detection of a chair object. (**a**) True positives; (**b**) misclassifications.

In Figure 21, examples of a correct detection and a misclassification of a chair object, displaying different stages of the process are shown.

In Figure 21a, on top, the adjusted depth image is observed; on the right, the thresholded image; in the lower right corner, the result of the segmentation process; and in the center, the image with the detected object. The name of the object is in the center of mass of the extracted contour. On the other hand, in Figure 21b, the chair could not be detected, because the legs could not be segmented from the depth image; because of this, in the lower right corner, the contours cannot be displayed.

In case of closets, 60 frames were taken, divided into 47 frames with different type of closets (specifically small closet and large closet) at different angles and 13 frames with other objects of the chosen environment. In Table 6, the confusion matrix with the results is shown. The analysis of the results shows that the developed classifier has a true positive rate of 63,83% and a specificity equal to 92.31%, as shown in Table 7.

**Figure 22:**
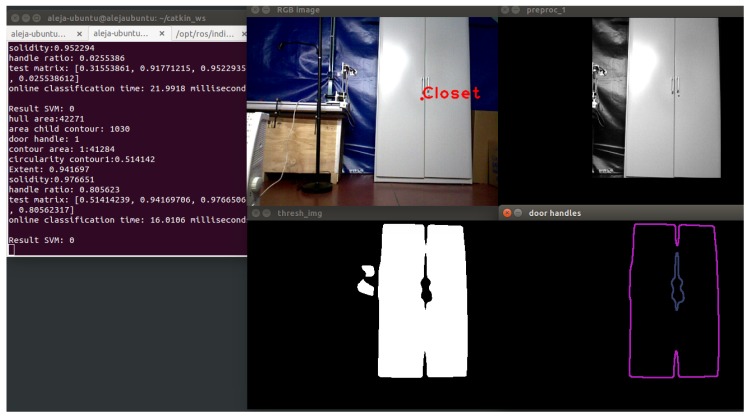
Example of true positives: Closet detection.

The model accuracy is 78.07%, with a 21.93% of misclassification rate. It was observed that at 3.60 m, the small closet was not detected. The system is able to detect small closets at a distance of 3.00 m. In Figure 22, a correct classification is shown.

The higher right corner shows the mask created in the initial segmentation. Then, the object is isolated, and using techniques of contour extraction, the biggest contour and the inside contour are obtained. The inside contour represents the door handles of the closet.

Additionally, the proposed system has been tested using a standard dataset, the NYU Depth Dataset V2 [25]. We selected RGB images of different closets. We compared the classification results obtained of our dataset and the NYU2 dataset, and we have attained good results in the detection of the closet objects. Table 8 and Table 9 show the results.

The model accuracy is 74.51%, with a 25.49% of misclassification rate. The results show only 3.56% less accuracy than using our own dataset.

On the other hand, in case of screens, Table 10 shows the confusion matrix with the results of the test. Table 11 shows the evaluations performed for binary classification.

**Figure 23:**
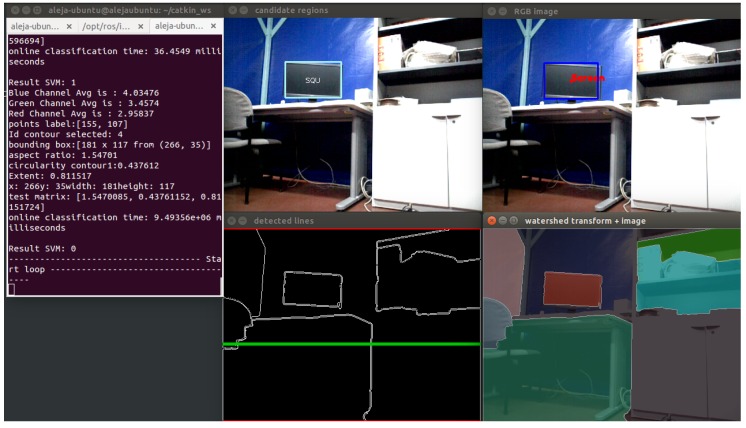
Example of true positives: Screen detection.

A total of 84 frames were taken for testing, divided into 60 frames with different types of screens (specifically computer screen and television screen) at different angles, and 20 frames with other objects of the selected environment. The results show that the developed classifier has a true positive rate of 60.94% and a specificity equal to 70%. The model accuracy is 65.47%, with a 34.53% misclassification rate. In Figure 23, a correct classification is shown. When the segmentation method works correctly and the objects in the scene can be separated better, the screens can be detected correctly.

After the preliminary tests, the following can be concluded: the best accuracy is for the closet object (78.07%), followed by chairs with 71.45% and, finally, the screens with the lowest percentage of 65.47%. Regarding the tests about the SVM parameters, the best performance of the proposed system was obtained with nu=0.2. The findings of these tests have been taken into account to improve the proposed system to perform the successive tests.

## 8. Implementation of Shape Descriptors

In this section, the results of applying the method to feature extraction based on shape descriptors in the proposed detection system are presented. For this test, the camera is integrated into the TurtleBot at a distance of 40 cm from the floor. Likewise, three SVM are used, one for each object to detect. RGB and depth images are employed to find the objects in the scene. The procedure consists of moving the TurtleBot by teleoperation around the area of the test and taking frames with the camera to analyze the results. During the test, 180 frames were taken for testing, divided into 57 frames with different types of closets (specifically, small closets and large closets) at different angles, 59 frames with chairs in different positions, 25 frames with screens in different angles and 39 frames with other objects selected from the environment. Table 12 shows the evaluations performed for binary classification.

**Figure 24:**
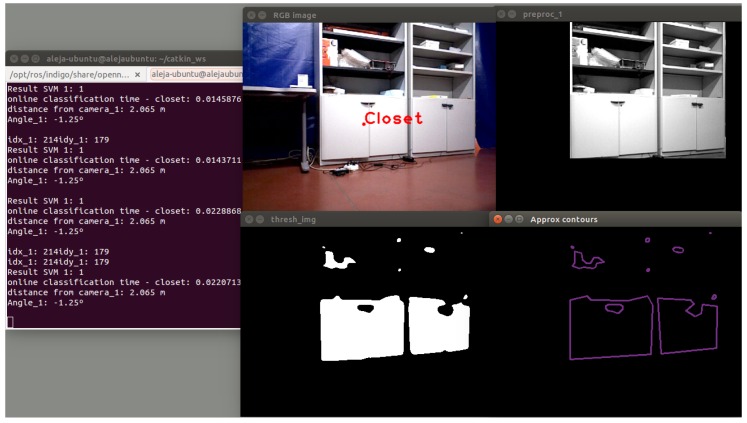
True positives using shape descriptors.

Analysis of the results shows that the developed classifier has a true positive rate of 66,10% as a high value for chairs and a specificity equal to 100% for closets. The model accuracy is better for closets (81.58%), followed by chairs and screen objects with 72.79% and 65.60%, respectively. The highest misclassification rate is for screens. In Figure 24, an example of the correct classification of the closet object is shown.

However, sometimes, the closets are not detected if the door handles are not found. In some cases, if the TurtleBot is very near the closet, in the segmentation process, only a part of the object is isolated, generating errors in the prediction process. A similar situation occurs with chairs: if the robot is very near the chairs, it is not able to detect them. Regarding the screens, the difficulty of finding features that distinguish them from other objects in the environment, which share similar geometric features, makes it the most difficult object to detect for the proposed system.

## 9. Implementation of Bag of Words

In this section, the results of applying the method to feature extraction based on bag of words in the proposed detection system are described. The idea is to treat image features as words. The SURF extractor and descriptor is used. The camera is integrated into the TurtleBot at a distance of 40 cm from the floor. A single SVM is used, with the same SVM parameters from the previous experiment. As input, RGB images are used to find the objects in the scene. In this test, any previous segmentation method is applied, in order to test the quality of the descriptors. Only for screens, a segmentation algorithm based on the selection of regions of interest (ROI) has been applied. In Table 13, the results of the evaluation can be seen.

A total of 75 frames were taken for testing, divided into 24 frames with different types of closets, 21 frames with chairs, 11 frames with screens at different angles and 19 frames with other objects of the selected environment. The results show that the highest model accuracy is for closets, with 85.42% followed by screens with 76.56% and chairs with 73.06%. The highest misclassification rate is for chairs with 26.94% and screens with 23.44%. Sometimes, if the robot perceives the chair back, the system is unable to detect it. In the case of the closets, when the robot is more than three meters away from the object, it cannot be detected.

## 10. Comparison of Features Extraction Methods

After the conducted tests, the results show that using descriptors combined with bag of words as the feature extraction technique, the model accuracy increases for the three selected objects, as follows, from 81.58% to 85.42% for closets, from 72.79% to 73.06% for chairs and from 65.90% to 76.56% for screens. The misclassification rate decreases in all of the objects, especially in the case of the screens from 34.10% to 23.44%. Regarding the rate of the true positives, the biggest increase is for closets, from 63.13% to 70.83%. The main reason for these results is the large number of descriptors extracted on each image that makes the classifier more robust.

On the other hand, despite the good results obtained using bag of words for object detection, it was not possible to determine the location of each object in the scene. This represents a problem considering that the objectives of this work not only include the detection of the selected objects, but also their locations in the original image, in order to have information available to contribute in navigation tasks, place categorization and semantic navigation. The detection using descriptors allows one to determine the presence or absence of objects in the scene, but not to know where they are. Furthermore, the training stage is slower than using shape information due to the higher number of features that are extracted.

## 11. Integration with a Navigation System

**Figure 25:**
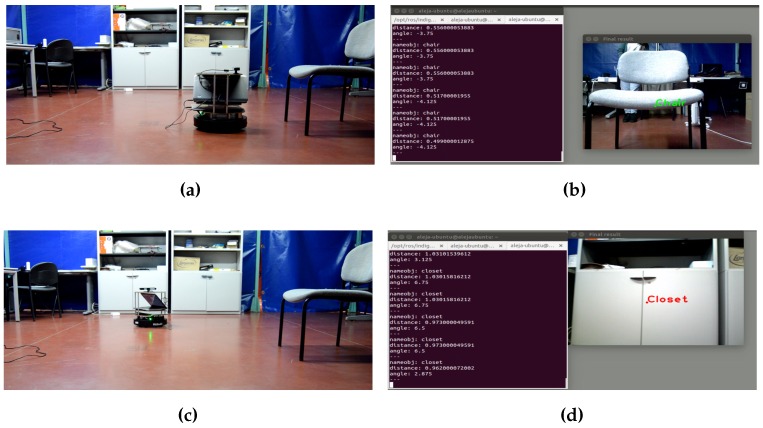
Results of the integration of both system into TurtleBot. (**a**) TurtleBot in front of the first target; (**b**) the point of view of the robot; (**c**) TurtleBot in front of the second target; (**d**) the view of the robot during the detection of the closet.

This last test consists of integrating the proposed detection system with a navigation system to evaluate the real-time functioning of both systems together. The model developed is categorized as a topological representation based on movements; this means that the relations between nodes have no geometrical meaning. In this integration test, the information perceived by the detection system is processed, and several common objects of a room are detected. Navigation is structured according to the position of those objects. The navigation system receives the information of the detected object (name, distance and orientation angle), and in the case that this information corresponds to the desired one for the event, the robot moves towards that object. For this test, a mobile robotic platform developed by [33] and the TurtleBot have been used. In Figure 25, the results of this experiment are shown.

With this test, it can be said that the process of integration has been completed successfully, and the navigation targets have been achieved.

## 12. Conclusions

In this work, a vision system has been proposed. This system is able to detect and locate objects in indoor environments, which contributes to improving the navigation of mobile robots serving as an entry to place recognition and high level tasks concerning semantic navigation. For this, the work has focused on the detection of three objects present in indoor environments: chairs, closets and screens. Likewise, the system has been integrated into a real mobile robot (TurtleBot), and a camera Xtion pro Live, which provides RGB and depth images, has been used.

To demonstrate the usefulness of the developed system, several experimental tests have been performed. Initially, individual tests for each type of object to refine some variables of the system were performed. From the results, it has been determined that the best accuracy is for the closet object, followed by chairs and screens. The findings of these tests have been taken into account to improve the proposed system to perform the successive tests.

Afterwards, experiments using shape descriptors and bag of words as feature extraction methods were done. The results show that applying the method to feature extraction based on bag of words in the proposed detection system, higher accuracy and a lower misclassification rate for all objects were obtained. The main reason for this is the large number of descriptors extracted on each image that makes the classifier more robust. This method allows one to determine the presence or absence of objects in the scene; nevertheless, it is very difficult to find where the detected object is in the image. Furthermore, the training stage is slower than using shape information due to the higher number of features that are extracted. All of the analysis performed suggests that the best option for the detection of selected objects consists of three SVM, using shape descriptors, such as the feature extraction method, taking into account the aims of this work.

On the other hand, a test to integrate the proposed detection system with a topological navigation system to evaluate the real-time functioning of both systems together has been performed. The systems have been incorporated into a real robotic platform (TurtleBot). According to the results, the integration process has been conducted successfully, and the navigation targets have been achieved.

Finally, future works will include the incorporating of semantic information and 3D information (point clouds) to develop a general segmentation method that allows better discrimination of all objects in the scene, improving each object model through feature extraction to see whether new combinations can further enhance the performance of the proposed system and integrating the developed system in a semantic navigation system.

## Figures and Tables

**Table 1:** Parameters of each SVM.

Parameters	Value
SVM type	One class
Kernel type	Linear
Nu	0.5
Gamma	0.50625
C	312.5
No. of iterations	100
Tolerance error	0.000001

**Table 2:** Possible results of the three SVMs.

Case	SVM 1	SVM 2	SVM 3	Filter Result
1	1	1	1	Closet
2	1	1	0	Closet
3	1	0	1	Closet
4	1	0	0	Closet
5	0	1	1	Chair
6	0	1	0	Chair
7	0	0	1	Screen
8	0	0	0	Undefined

**Table 3:** The confusion matrix for the two-class classification problem.

	Positive (Predicted)	Negative (Predicted)
**Positive (actual)**	a	b
**Negative (actual)**	c	d

**Table 4:** Confusion matrix: Chair detection.

%	Chair	Undefined
**Chair**	65.12%	34.88%
**Undefined**	22.22%	77.78%

**Table 5:** Evaluations: Chair detection.

Evaluations	%
Model accuracy	71.45%
Misclassification rate	28.55%
True positive rate	65.12%
True negative rate	77.78%

**Table 6:** Confusion matrix: Closet detection.

%	Closet	Undefined
**Closet**	63.83%	36.17%
**Undefined**	7.69%	92.31%

**Table 7:** Evaluations: Closet detection.

Evaluations	%
Model accuracy	78.07%
Misclassification rate	21.93%
True positive rate	63.83%
True negative rate	92.31%

**Table 8:** Confusion matrix: closet detection with the NYU2 dataset.

%	Closet	Undefined
**Closet**	66.67%	33.33%
**Undefined**	17.65%	82.35%

**Table 9:** Evaluations: closet detection with the NYU2 dataset.

Evaluations	%
Model accuracy	74.51%
Misclassification rate	25.49%
True positive rate	66.67%
True negative rate	82.35%

**Table 10:** Confusion matrix: Screen detection.

%	Screen	Undefined
**Screen**	60.94%	39.06%
**Undefined**	30.00%	70.00%

**Table 11:** Evaluations: Screen detection.

Evaluations	%
Model accuracy	65.47%
Misclassification rate	34.53%
True positive rate	60.94%
True negative rate	70.00%

**Table 12:** Evaluation of the proposed system applying shape descriptors.

Evaluations	Closets	Chairs	Screens
Model accuracy	81.58%	72.79%	65.90%
Misclassification rate	18.42%	27.21%	34.10%
True positive rate	63.13%	66.10%	60.00%
True negative rate	100.00%	79.49%	71.79%

**Table 13:** Evaluation of the proposed system using bag of words.

Evaluations	Closets	Chairs	Screens
Model accuracy	85.42%	73.06%	76.56%
Misclassification rate	14.58%	26.94%	23.44%
True positive rate = sensibility	70.83%	61.90%	63.64%
True negative rate = specificity	100.00%	84.21%	89.47%
